# Outcomes of BRCA pre-implantation genetic testing according to the parental mutation origin: a cohort study

**DOI:** 10.1186/s12958-023-01180-9

**Published:** 2024-01-03

**Authors:** Ilana Weizel, Tal Shavit, Yulia Shuli, Chana Adler Lazarovich, Rivka Halevi, Tal Ben Ari, Shira Yaacobi-Artzi, Yaakov Bentov, Baruch Feldman, Anat Hershko Klement

**Affiliations:** 1 The IVF Unit, Department of Obstetrics and Gynecology, Hadassah Mount Scopus- Hebrew University Medical Center, Mt Scopus, Jerusalem, 9112001 Israel; 2 The IVF unit, Assuta Medical Center, Tel Aviv, Israel; 3 Faculty of Medicine, Ben Gurion University of the Negev, Beer Sheva, Israel; 4 The Unit for Medical Genetics, Assuta Medical Center, Tel Aviv, Israel; 5 Department of Obstetrics and Gynecology, Faculty of Medicine, Hebrew University of Jerusalem, Jerusalem, Israel

**Keywords:** BRCA gene, Preimplantation genetic diagnosis, Preimplantation embryo development, In vitro fertilization, Inheritance

## Abstract

**Background:**

The process of gamete formation and early embryonic development involves rapid DNA replication, chromosome segregation and cell division. These processes may be affected by mutations in the *BRCA*1/2 genes. The aim of this study was to evaluate BRCA mutation inheritance and its effect on early embryonic development according to the parental origin of the mutation. The study question was approached by analyzing in vitro fertilization cycles (IVF) that included pre-implantation testing (PGT-M) for a BRCA gene mutation.

**Methods:**

This retrospective cohort study compared cycles of pre-implantation genetic testing for mutations (PGT-M) between male and female patients diagnosed with BRCA 1/2 mutations (cases), to a control group of two other mutations with dominant inheritance (myotonic dystrophy (MD) and polycystic kidney disease (PKD)). Results were compared according to mutation type and through a generalized linear model analysis.

**Results:**

The cohort included 88 PGT-M cycles (47 BRCA and 41 non-BRCA) among 50 patients. Maternal and paternal ages at oocyte retrieval were comparable between groups. When tested per cycle, FSH dose, maximum estradiol level, oocytes retrieved, number of zygotes, and number of embryos available for biopsy and affected embryos, were not significantly different among mutation types. All together 444 embryos were biopsied: the rate of affected embryos was comparable between groups. Among BRCA patients, the proportion of affected embryos was similar between maternal and paternal mutation origin (*p* = 0.24). In a generalized linear model analysis, the relative oocyte yield in maternal BRCA patients was significantly lower (0.7, as related to the non BRCA group)(*p* < 0.001). Zygote formation and blastulation were not affected by the BRCA gene among paternal cases (P = 0.176 and P = 0.293 respectively), nor by paternal versus maternal BRCA carriage (P = 0.904 and P = 0.149, respectively).

**Conclusions:**

BRCA PGT-M cycles performed similarly compared to non-BRCA cycles. Inheritance rate and cycle parameters were not affected by the parental origin of the mutation.

## Background

The process of gamete formation and early embryonic development involves rapid DNA replication, chromosome segregation and cell division. These processes may be affected by mutations in the *BRCA*1/2 genes. *BRCA* is a cell cycle checkpoint and DNA repair gene [1]. The BRCA1/2 genes are crucial for the repair of double strand DNA breaks by homologous recombination [2]. *BRCA1* is also involved in chromatin remodeling and transcriptional control which contribute to its role as a tumor suppressor gene [3]. BRCA genes also participate in the repair and maintenance of chromosome telomeres [4].

The BRCA genes have additional crucial roles. *BRCA1* is involved in meiotic inactivation of sex chromosomes which functions in the silencing of non-homologous regions of sex chromosomes [5]. The related process of meiotic silencing of unsynapsed chromatin also requires *BRCA1* and operates in both male and female germ cells [6]. The *BRCA2* gene has a role as an essential mediator of homologous recombination during spermatocyte meiosis [7].

Several studies have noted the detrimental effects of BRCA mutations on female reproduction and early embryo development. These included reports of a reduced response to ovarian stimulation [2], a stronger deleterious effect of gonado-toxins [3], younger age at menopause [4] and increased embryonic susceptibility to ROS formation among carriers of BRCA mutations [5]. However, other groups failed to show similar effects of BRCA mutation carriers on reproduction [6, 7]. A recent study by Drechsel and colleagues [8] evaluated the effect of a BRCA mutation on ovarian reserve status by measuring AMH and ACF levels, and ovarian response in BRCA mutation carriers. No significant differences were found between BRCA mutation carriers and healthy controls undergoing the same type of assisted reproduction treatment.

The potential detrimental effects of BRCA mutations may be related to the parental origin of the mutated gene (male/female), the type of gene (*BRCA 1/2*) and the stage of embryonic development assessed. As a result of differential demethylation and lack of DNA repair mechanisms in the sperm, a paternal mutation in the BRCA genes may have a stronger negative effect. On the other hand, the impact of these DNA repair gene mutations may not be apparent during the early stages of embryonic development.

The aim of this study was to evaluate BRCA mutation inheritance and its effect on early embryonic development according to the parental origin of the mutation. We studied these questions in an IVF setting by analyzing in vitro fertilization cycles (IVF) that included pre-implantation testing (PGT-M) for a BRCA gene mutation compared to PGT-M cycles involving 2 other mutations with dominant inheritance (myotonic dystrophy (MD) and polycystic kidney disease (PKD).

## Materials and methods

This retrospective, cohort study included IVF PGT-M cycles evaluated by the Genetics Laboratory in Assuta Tel Aviv Medical Center from 2018 to 2022. The study group included PGT-M cycles performed for male and female patients diagnosed with *BRCA 1/2* mutations (cases). Cases were compared to PGT-M cycles tested for other mutations with dominant inheritance (MD or PKD) from either the male or female partner (controls).

### Ethics

The study was approved by the Ethics Committee of Assuta Tel Aviv Medical Center (approval number 0092 − 21 ASMC). All patients a genetic consultation prior to a PGT cycle and therefore all patients who were eventually included in this study expressed their wish to perform an IVF PGT-M cycle for this indication. Written informed consent was not required for this retrospective study.

### Pre-implantation genetic testing

The oocytes destined for ICSI or for standard insemination were checked for fertilization 16 to 18 h after the procedure. The zygotes were then transferred into individual wells with 25 µl of pre-equilibrated single medium (SAGE 1-STEP, Origio, Trumbull, CT, USA or GLOBAL TOTAL, LifeGlobal, Paramus, NJ, USA) under mineral oil and cultured until day5 or 6 in a time-lapse system incubator (EmbryoScope, Vitrolife, Sweden), under standard incubation conditions (37 °C, 6% CO_2_ and 5% O_2_). Embryos were evaluated for cleavage stage (day 2/3) based on recording the number and symmetry of blastomeres, and the percentage of fragmentation. Blastocysts (day 5/6) were evaluated using a previously described scoring system [9]. Briefly, the blastocysts were classified as early (1–2 Gardner scale) or expanding (3–6 Gardner scale). For blastocysts graded as 3–6, the development of the inner cell mass and the trophectoderm were assessed. The inner cell mass was designated as: (A) tightly packed, many cells, (B) loosely grouped, several cells, or (C) very few cells. The trophectoderm was described as: (a) many cells forming a cohesive epithelium, (b) few cells forming a loose epithelium, or (c) very few, large cells. Only embryos of good morphology were biopsied (grade A or B). Biopsies were performed on day 5 or on the morning of day 6 depending on the grade of the expanded blastocyst (3–6, i.e., full blastocysts onward). Biopsies conducted on day 3 were performed at the 6–8 cell stage. Before biopsy, the cleavage stage embryos were pre-incubated for 10–15 min in Ca^+ 2^/Mg^+ 2^ free bicarbonate-buffered medium (Vitrolife, Sweden) to loosen cell-to-cell adhesions. Biopsies were undertaken using an inverted microscope (Diaphot 300, Nikon, Japan) equipped with a warming stage and micromanipulation system (Narashige, Japan). Laser technology (ZILOS-tk, Hamilton Thorne, Beverly, MA, USA) was used to dissect the zona pellucida, and one blastomere or 5 to 6 trophectoderm cells were pulled gently away from cleavage stage embryos/blastocysts. Before biopsy, the blastomeres were checked for the presence of nuclei. Each blastomere and trophectoderm cell was routinely washed prior to their transfer to the PCR tube, to ensure a pure sample. Moreover, a sample from the last drop of the washing medium was also collected in a different PCR tube and transferred for molecular analysis as a control, to detect contamination. After the procedure, the biopsied embryos were placed in separate numbered dishes with pre-equilibrated single medium to ensure easy identification of embryos post-diagnosis.

### Molecular diagnosis

Establishing the haplotype was a necessary step in the preparation for PGT. Several informative microsatellite markers were selected for each patient in preparation for PGT. At least two informative polymorphic short tandem repeats were linked for each family on either side of the mutant allele. Primers suitable for multiplex PCR were carefully designed for each marker, and the diagnostic protocol was examined using DNA samples of appropriate family members who were carriers or non-carriers of the specific familial mutant allele. Each case was pre-validated in a model specifically designed for each family. Validation was achieved by employing genomic DNA samples for haplotyping and highly diluted DNA samples for pre-PGT validation, mimicking single-cell molecular testing.

Maternal and paternal DNA samples were always included in each case; therefore, these informative markers could differentiate the maternal and paternal contribution of alleles to all normal or abnormal embryos. The genetic constitution of each normal embryo is composed of one maternal and one paternal allele. An embryo with unequal parental contribution is easily detectable, while the origin of the extra allele is straightforwardly diagnosed by the pre-validated markers. Using multiple informative markers in all cases allows us to diagnose all embryos with uniparental disomy [10].

### Data analysis

All data were analyzed using SPSS for Windows, version 24.0 (IBM Corp., Armonk, NY, USA). As the continuous variables were not normally distributed, we used descriptive statistics including the median values and non-parametric tests for the comparison of continuous variables among independent groups (Mann-Whitney U test). Chi-square test or Fisher’s exact test was used to compare rates and proportions, each when appropriate. The BRCA PGT cycles were compared to non-BRCA cycles (MD and PKD). Since some of the patients had more than one cycle, we also accounted for the total contribution of each patient and performed a generalized linear model analysis with a log link function, utilizing the natural logarithm (Ln) with the mathematical base e :When analyzing the oocyte yield, the model used the number of cycles (Ln cycles) as the offset variable. In the analysis of zygotes and biopsied embryos (representing the blastulation potential), the oocytes (Ln-oocytes) and zygotes (Ln-zygotes) were defined as the offset variables. All P values were two-tailed and considered significant at less than 0.05.

## Results

**Fig. 1:**
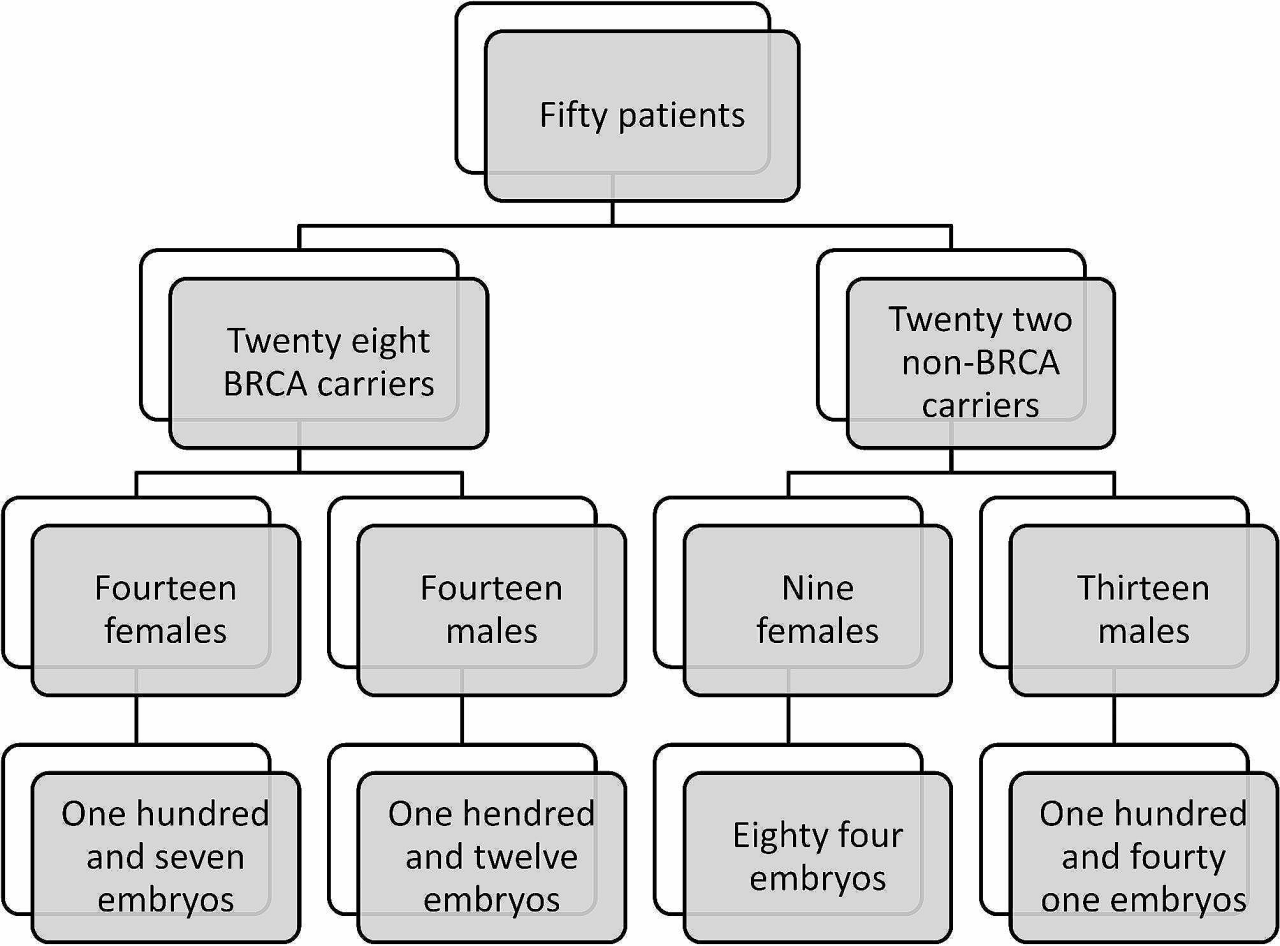
Study cohort by mutation type and parental origin

This study included 88 PGT-M cycles from 50 patients (Fig. 1), of which 47 cycles were performed for 28 BRCA patients (14 maternal, 14 paternal), and 41 cycles were non-BRCA PGT cycles (24 PKD, 17 MD) performed by 22 control patients (9 maternal, 13 paternal). The maternal and paternal ages at oocyte retrieval, and cycle performance were comparable between BRCA carriers and controls. Importantly, the women in both groups were of similar ages (P = 0.33; Table 1). When tested according to cycle parameters, including FSH dose, maximum estradiol level, oocytes retrieved, mature oocytes, number of zygotes, as well as number of embryos available for biopsy and affected embryos, none were statistically significant when compared across mutation types (Table 1).

**Table 1:** Paternal and maternal ages at oocyte retrieval and cycle performance according to mutation type (data are presented per cycle)

Parameter	BRCA PGT cycles (n = 47)		Non-BRCA PGT cycles (n = 41)		*P*- value
	Mean ± SD	Median	Mean ± SD	Median	
Maternal age (years)	32.4 ± 3.9	31	33.1 ± 5.4	33	0.33
Paternal age (years)	33.9 ± 4.6	34	35.0 ± 5.5	35	0.21
Maximum estradiol level (pmol/L)	10248.7 ± 6339.2	8935	12585.1 ± 7640.8	10,620	0.13
FSH dose (IU)	3721.71 ± 4082.8	2293.5	3018.64 ± 1535.3	2587.5	0.41
Number of oocytes retrieved	15.7 ± 7.5	15	20.7 ± 3.6	18	0.14
Number of mature eggs	11.7 ± 5.7	10.5	15.1 ± 10.3	12	0.28
Number of zygotes	8.4 ± 4.7	8	10.4 ± 6.9	8	0.24
Embryos available for biopsy	5.1 ± 2.9	5	5.8 ± 4.0	4	0.68
Affected embryos	2.5 ± 2.3	2	2.8 ± 2.0	2	0.3

Altogether, 444 embryos were biopsied; 83.3% on day 5. We found the total fraction of affected embryos to be comparable between cases and controls: 0.469 of the embryos in the BRCA group were affected, compared to 0.486 in the non-BRCA group (p = 0.7). Specifically, among BRCA patients, the proportion of affected embryos was similar in maternal (mean 0.53 ± 0.20, median 0.5) versus paternal mutation origin (mean 0.40 ± 0.22, median 0.5; P = 0.24).

Since patients contributed different numbers of cycles, we analyzed the data correcting for the number of PGT cycles and accounting for the number of available oocytes and zygotes (Table 2). In a generalized linear model analysis with a log link function, exp (β) reflects the proportion between the groups : compared to the non-BRCA maternal cases, The oocyte yield in maternal BRCA patients was significantly lower (0.7 as related to the non BRCA group, or in reciprocal calculation 1.396 more oocytes were retrieved in non BRCA cases)(*P* < 0.001). Zygote formation and blastulation were not affected by the BRCA gene among paternal cases (P = 0.176 and P = 0.293 respectively), nor by paternal versus maternal BRCA carriage (P = 0.904 and P = 0.149, respectively; Table 2).

**Table 2:** Oocyte yield, zygote formation and available embryos by type of mutation and parental origin – model summary, corrected for number of cycles (Ln-cycles) and available oocytes/zygotes (Ln-oocytes and Ln-zygotes)

Patients in the model	Offset	Outcome	β	exp (β)	Standard error	*p*-value
Brca compared to non-BRCA **female carriers only**	Ln-cycles	Number of oocytes	-0.334	0.716	0.079	< 0.001
Brca compared to non-BRCA **male carriers only**	Ln-oocytes	Zygotes	0.129	1.137	0.095	0.176
Brca compared to non-BRCA: **male carriers only**	Ln-zygotes	Embryos biopsied	0.136	1.146	0.120	0.293
BRCA male carriers compared to BRCA females	Ln-oocytes	Zygotes	0.013	1.013	0.105	0.904
BRCA male carriers compared to BRCA females	Ln-zygotes	Embryos biopsied	-0.199	0.819	0.137	0.149

## Discussion

The results of this study show that BRCA PGT-M had similar outcomes compared to non-BRCA PGT-M cycles in terms of fertilization rate, blastulation, and fraction of affected embryos. In our cohort, these parameters were not influenced by the parental origin of the mutation. We found a significantly lower oocyte yield in female BRCA-carrier patients, possibly related to a previously suggested mechanism of accumulated damage to oocyte DNA due to repair failure [3].

The completion of embryogenesis relies on the activation of both maternal and paternal genes, while the differential demethylation of the parental genomes has the potential to regulate the early development of preimplantation embryos [11]. Embryonic genome activity can be detected 9–10 h after fertilization. Earlier embryonic signals derived from maternal mRNA are termed the maternal legacy [12]. The stored mRNA can guide development under maternal control until embryonic genome activity. The degradation of particular inherited mRNAs may regulate the timing of embryonic genome activity [13]. Recent studies have suggested that there are three distinct waves of embryonic genome activation [14] occurring at the 2-cell stage, 4-cell stage, and 8–10 stage. The final wave at the 8–10 cell stage is associated with the highest level of transcriptional activity [14]. A recent paper suggested that transcription is initiated soon after fertilization, during meiotic progression and gamete reprogramming, which should illuminate mechanisms that coordinate chromatin remodeling and transcription complex assembly [15]. In cases of pathogenic mutations in non-imprinted genes, the different levels of mutant and normal transcripts available for translation in the early-stage embryo will be determined by the parental origin of the variant in the embryo [11]. Due to different demethylation rates of the parental genomes when the mutation is paternally inherited, it may result in a greater transcription potential for the paternal genome around day 3 [11]. As for repair mechanisms in the embryos, DNA repair differs throughout embryogenesis [16] and only some mutations in repair genes delay embryonic development in the very early stages of development.

Changes in the methylation patterns of *BRCA 1* have been reported in early developing preimplantation embryos [11]. Therefore, embryos that inherited pathogenic variants of *BRCA 1 or 2* may express these genes differently according to the involved methylation wave. A study by Tulay et al. [11] reported slower embryonal development when the mutation was inherited from the father, compared to those inheriting the same mutation from the mother. Our results do not support that finding.

We did not find differences in embryonic performance in a PGT-M culture environment in embryos from BRCA patients compared to non-BRCA, or for paternal versus maternal mutation origin. A detrimental biological behavior arresting most of the embryos with ‘paternal’ inheritance would have been reflected in our cohort as a reduced biopsy potential on day 5 in the paternal BRCA group. Instead, we found a preserved embryogenesis potential, which may be related to a reduced expression of BRCA mutation in the growing embryo through gradual demethylation [17].

Interestingly, reports addressing the effects of parent-of-origin on disease development reported that paternal inheritance of BRCA resulted in an earlier age of diagnosis of breast and ovarian cancer [7] and that the risk of developing breast cancer was modestly higher in women with a paternally-inherited *BRCA1* mutation compared to maternally-inherited BRCA1 mutation, but not for women with a *BRCA2* mutation [18].

Previous studies that focused on ovarian reserve and oocyte yield in BRCA carriers reported reduced reproductive potential, and compromised performance of cryopreservation strategies among BRCA-mutation breast cancer patients [19]. Lambertini et al. found that female BRCA carriers had lower median AMH levels, needed higher doses of gonadotropins and had fewer oocytes [2]; similar to our cohort. Oktay et al. also reported poorer response to ovarian stimulation in patients with *BRCA* mutations [4].They hypothesized that deficient DNA repair in patients with *BRCA* mutations may result in oocytes that are more prone to DNA damage. An oocyte may reside in the ovary for decades prior to its ovulation. During these years, accumulated DNA damage may be severe [20]. The significance of the damage may only be recognized once the follicle containing a certain oocyte is recruited and DNA replication commences. Research in nonreproductive cell types demonstrate that when DNA damage is severe and cannot be repaired, apoptotic pathways are activated [21, 22]. Thus, oocytes with deficient *BRCA* function may be prematurely eliminated by a similar mechanism, resulting in early depletion of egg reserve and even primary ovarian insufficiency.

In contrast, other studies have shown that BRCA carriers exhibit comparable ovarian reserves and responses to ovarian stimulation. For instance, when BRCA carriers were compared to women undergoing elective egg freezing, the carriers had preserved follicular count and cryopreserved oocytes, as compared with non-carriers [6]. Another retrospective cohort study compared BRCA mutation carriers undergoing IVF for PGT or fertility preservation to non-BRCA patients, matched by age, protocol and cancer disease status; Stimulation length and total stimulation dose were comparable between carriers and noncarriers, as were the oocyte yield, number of zygotes, fertilization rates and conception rates [7].

The current study had certain limitations which should be acknowledged: Although large in terms of previous reports, the sample was limited and too small to enable separation of the *BRCA1* and *BRCA2* variants. Further research is warranted to address these limitations and expand our knowledge in these areas, as well to investigate additional aspects such as morphology or euploidy rates. The strengths of the study lies in its design, enabling a comparison between paternal and maternal origins within BRCA carriers and between BRCA carriers and other mutations. Additionally, this study provides valuable information, contributing to the limited existing knowledge in the field. By analyzing the data both in relation to the treatment cycle and in relation to individual patient performance, a more comprehensive understanding of the factors influencing outcomes can be achieved.

## Conclusion

We observed that the parental origin of the BRCA mutation did not appear to have an impact on early embryonic development. These findings suggest that BRCA PGT-M cycles are comparable to PGT-M cycles of other inherited conditions and provide reassurance to BRCA carrier patients considering IVF for PGT-M.

This study also highlights the potential of IVF PGT cycles to assess the biological performance of genes involved in early embryogenesis.

## Data Availability

Upon request.

## Abbreviations

IVF: In vitro fertilization cycles
PGT: Pre-implantation testing
PGT M: Pre-implantation testing for mutation
MD: Myotonic dystrophy
PKD: Polycystic kidney disease
mRNA: Messenger RNA
AMH: Anti mullerian hormone
